# Unique transition of yielding mechanism and unexpected activation of deformation twinning in ultrafine grained Fe-31Mn-3Al-3Si alloy

**DOI:** 10.1038/s41598-021-94800-6

**Published:** 2021-08-05

**Authors:** Yu Bai, Hiroki Kitamura, Si Gao, Yanzhong Tian, Nokeun Park, Myeong-heom Park, Hiroki Adachi, Akinobu Shibata, Masugu Sato, Mitsuhiro Murayama, Nobuhiro Tsuji

**Affiliations:** 1 School of Materials Science and Engineering, Dalian University of Technology, No.2 Linggong Road, Ganjingzi District, Dalian, 116024 People’s Republic of China; 2 Department of Materials Science and Engineering, Kyoto University, Yoshida-honmachi, Sakyo-ku, Kyoto, 606-8501 Japan; 3 Elements Strategy Initiative for Structural Materials (ESISM), Kyoto University, Yoshida-honmachi, Sakyo-ku, Kyoto, 606-8501 Japan; 4 Key Laboratory for Anisotropy and Texture of Materials (Ministry of Education), School of Materials Science and Engineering, Northeastern University, Shenyang, 110819 People’s Republic of China; 5 Department of Materials Science and Engineering, Yeungnam University, Gyeongbuk, 38541 Korea; 6 Department of Materials and Synchrotron Radiation Engineering, Graduate School of Engineering, University of Hyogo, Himeji, 671-2280 Japan; 7 National Institute for Materials Science (NIMS), 1-2-1 Sengen, Tsukuba, 305-0047 Japan; 8 Japan Synchrotron Radiation Research Institute (JASRI), Sayo-gun, Hyogo, 679-5198 Japan; 9 Institute for Materials Chemistry and Engineering, Kyushu University, Kasuga, Fukuoka, 816-8580 Japan; 10 Department of Materials Science and Engineering, Virginia Tech, Blacksburg, VA 24061 USA

**Keywords:** Materials science, Structural materials, Metals and alloys

## Abstract

Tensile mechanical properties of fully recrystallized TWIP steel specimens having various grain sizes (*d*) ranging from 0.79 μm to 85.6 μm were investigated. It was confirmed that the UFG specimens having the mean grain sizes of 1.5 μm or smaller abnormally showed discontinuous yielding characterized by a clear yield-drop while the specimens having grain sizes larger than 2.4 μm showed normal continuous yielding. In-situ synchrotron radiation XRD showed dislocation density around yield-drop in the UFG specimen quickly increased. ECCI observations revealed the nucleation of deformation twins and stacking faults from grain boundaries in the UFG specimen around yielding. Although it had been conventionally reported that the grain refinement suppresses deformation twinning in FCC metals and alloys, the number density of deformation twins in the 0.79 μm grain-sized specimen was much higher than that in the specimens with grain sizes of 4.5 μm and 15.4 μm. The unusual change of yielding behavior from continuous to discontinuous manner by grain refinement could be understood on the basis of limited number of free dislocations in each ultrafine grain. The results indicated that the scarcity of free dislocations in the recrystallized UFG specimens changed the deformation and twinning mechanisms in the TWIP steel.

## Introduction

High-manganese austenitic steels, having austenite single phase with face-centered cubic (FCC) structure at ambient temperature, have attracted a great attention as one of the advanced high strength steels for next-generation automotive applications, because of their good balance of high strength and large ductility. The superior mechanical properties of high-manganese austenitic steels are considered to be attributed to deformation twinning frequently formed during plastic deformation, so that they are also known as twinning induced plasticity (TWIP) steels^1–10^.

However, the low yield strength of high-Mn austenitic TWIP steels due to their FCC structure is a weak point for practical application. Based on the Hall–Petch relationship^11^, grain refinement is a possible approach to improve the yield strength without changing chemical compositions in metallic materials. Severe plastic deformation (SPD) processes^12–14^ are the promising methods widely used to achieve ultrafine grained (UFG) microstructures with average grain sizes smaller than 1 μm, and the UFG materials produced by SPD show very high strength. Meanwhile, the UFG microstructures produced by SPD have deformed characteristics, since the mechanism of grain refinement by SPD is understood in terms of the grain subdivision^15–19^ during plastic deformation^20^. Thus the SPD processed UFG microstructures involve the high dislocation densities which limit additional strain-hardening after macroscopic yielding and result in small uniform elongation owing to the early plastic instability^21–23^. By now, thermomechanical processes have been applied on typical TWIP steels to improve their yield strength by grain refinement^24–27^. Fully recrystallized microstructures were obtained, and the minimum grain sizes achieved in those studies were 1.3–3 μm, which were, however, still in the conventional coarse-grained regime. The yield strength was fairly improved by the grain refinement and yielding occurred in the continuous manner, which was a typical yielding behavior in FCC metals. On the other hand, we have recently succeeded in fabricating fully recrystallized UFG microstructures in high-Mn austenitic steels simply by conventional cold-rolling and subsequent annealing without SPD^28,29^. The fully recrystallized UFG high-Mn steels managed both high strength and large tensile ductility^28–31^. It was noteworthy that the fully recrystallized UFG high-Mn steels exhibited very high yield strength as expected and unexpectedly discontinuous yielding accompanied with yield-drop phenomena in spite of their FCC structure.

It is well known that coarse-grained metallic materials having FCC structure usually exhibit continuous yielding, whereas metals having body-centered cubic (BCC) structure (typically iron and carbon steels) show discontinuous yielding accompanying with yield-drop phenomena due to locking and unlocking of dislocations by interstitial solute elements, such as carbon and nitrogen^32^. However, it has been reported that UFG metals with recrystallized structures show discontinuous yielding characterized by obvious yield-drop regardless of the crystal structures and alloy systems, such as FCC metals and alloys like pure Al^33^, pure Cu^34,35^, high-Mn austenitic steels^28^, Ni-40Co alloy^36^, and even high/medium entropy alloys^37^, as well as hexagonal close packed (HCP) metals like pure Ti^38^ and Mg alloys^39,40^. Although ultra-low carbon interstitial free (IF) steels are a kind of steels composed of ferrite single phase with BCC structure, they usually show continuous yielding because interstitial carbon and nitrogen atoms are fixed as carbides and nitrides of Ti and/or Nb^41^. However, when the average grain size is reduced below about 1–2 μm, even IF steels show the discontinuous yielding characterized by yield-drop and subsequent Lüders deformation^42^. These results indicate that the discontinuous yielding is a unique mechanical response universally occurring in UFG materials.

Nevertheless, detailed characteristics and mechanism of the discontinuous yielding accompanied with yield-drop in UFG metals including high-Mn steels have not been clarified yet. In the present study, we have tried to clarify the nature of discontinuous yielding in fully recrystallized high-Mn austenitic TWIP steel through systematic investigations using digital image correlation (DIC) during tensile deformation, electron channeling contrast imaging (ECCI) in a scanning electron microscope (SEM), and in-situ synchrotron radiation X-ray diffraction during tensile tests.

## Results

### Microstructure observations

**Figure 1:**
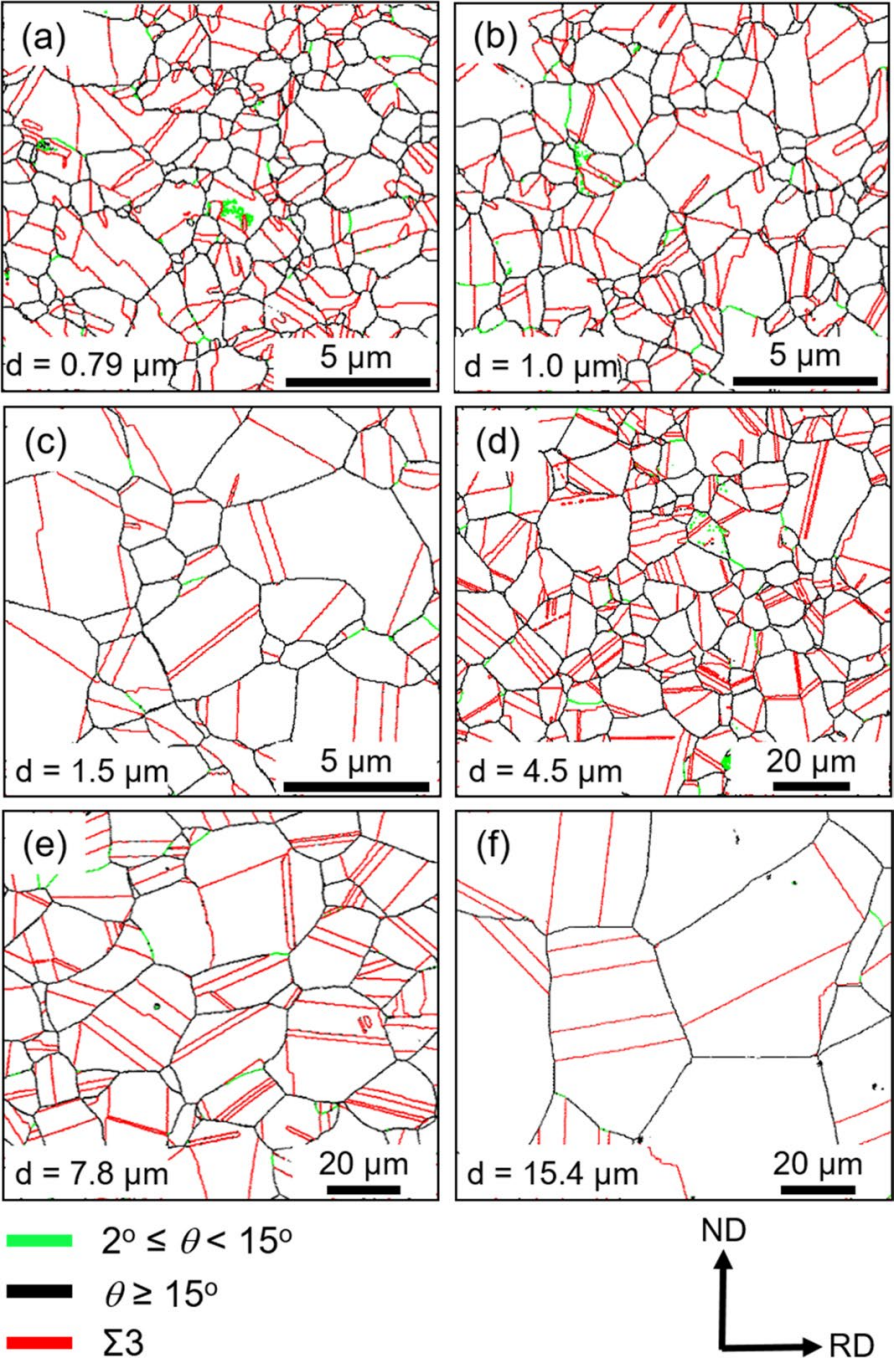
EBSD grain boundary maps of the specimens fabricated by the cold-rolling and subsequent annealing process: (**a**) Annealed at 700 °C for 300 s, (**b**) 750 °C, 300 s, (**c**) 800 °C, 300 s, (**d**) 900 °C, 300 s, (**e**) 950 °C, 300 s, and (**f**) 950 °C, 900 s. The green, black and red lines represent low-angle boundaries, high-angle boundaries and Σ3 twin boundaries, respectively. The high-angle boundaries were determined as boundaries with misorientations (θ) larger than 15° (15° ≤ θ), and low-angle boundaries with misorientations between 2° and 15° (2° ≤ θ < 15°), respectively. ND and RD indicate the normal direction and rolling direction of the sheets, respectively.

Figure 1 shows representative grain boundary maps obtained by SEM electron back-scattering diffraction (EBSD) measurements of the 31Mn-3Al-3Si TWIP steel specimens cold-rolled and annealed at various temperatures for different periods. The grain boundary maps showed that all the specimens were composed of fully recrystallized equiaxed grains surrounded mostly by high-angle boundaries. The fraction of annealing twin boundaries was around 50% in all cold-rolled and annealed specimens. The mean grain sizes (*d*) including annealing twin boundaries ranged from 0.79 μm to 85.6 μm and increased with increasing the annealing temperature or time. The specimens with the mean grain sizes of 0.79 μm, 4.5 μm and 15.4 μm were picked up for detailed investigations.

### Tensile properties

**Figure 2:**
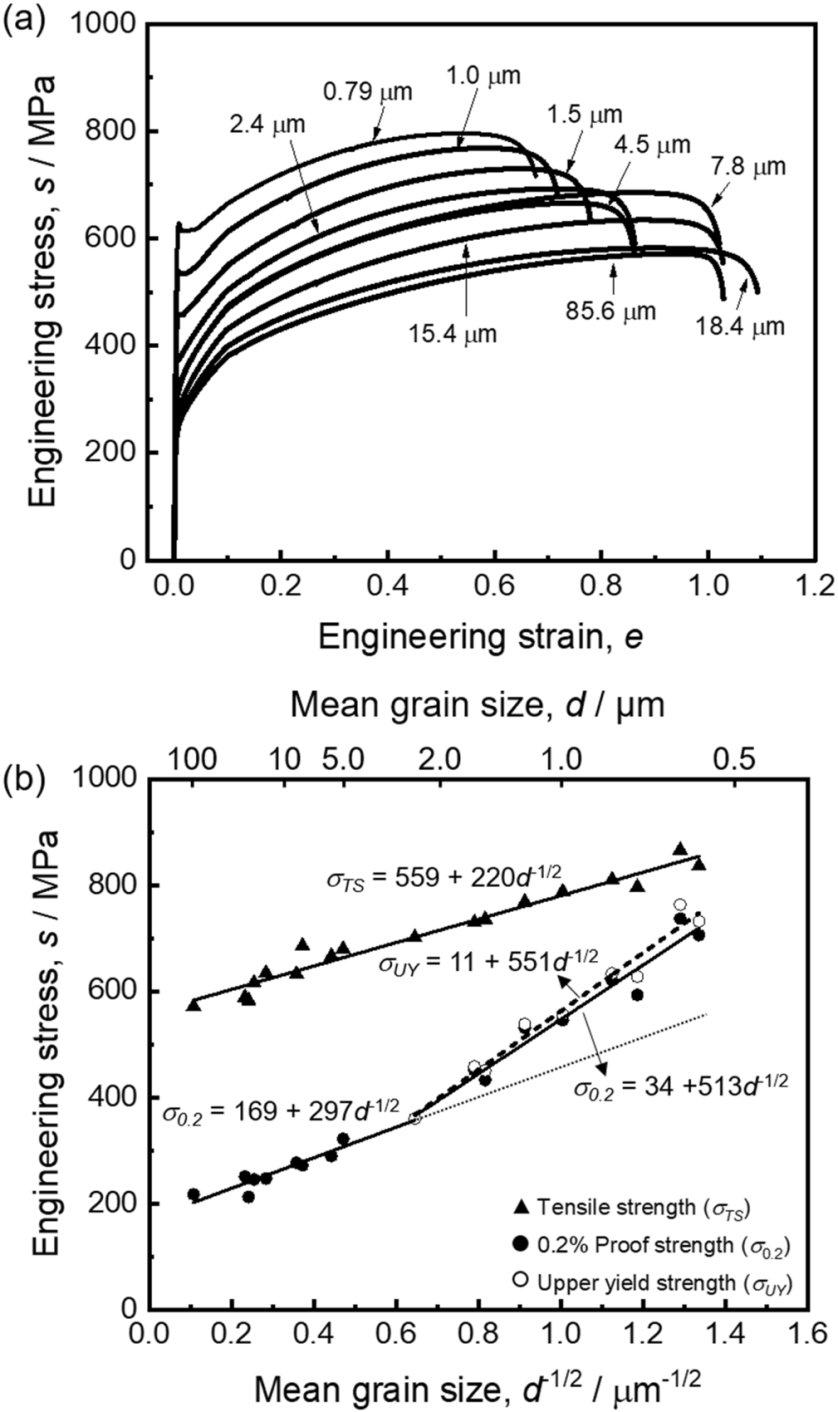
(**a**) Engineering stress–strain curves of the Fe-31Mn-3Al-3Si specimens with various recrystallized grain sizes. The mean grain sizes are indicated in the figure. (**b**) Hall–Petch plots of the yield strength and tensile strength as a function of minus square of the mean grain size.

Figure 2(a) shows engineering stress–strain curves of the specimens with various recrystallized mean grain sizes, obtained from the tensile test at room temperature. Yield and tensile strengths increased with decreasing the grain size. It was confirmed that the specimens with the mean grain sizes smaller than or equal to 1.5 μm showed discontinuous yielding characterized by clear yield-drop, while the specimens with the mean grain sizes larger than 2.4 μm exhibited typical continuous yielding. Tensile elongation somehow decreased with the grain refinement below 5 μm. However, the UFG specimens having the mean grain sizes smaller than 1 μm still showed large elongation over 60%, which was totally different from the UFG ferritic steels exhibited discontinuous yielding and limited tensile elongation of a few percent due to early plastic instability^21^. The large uniform elongation in the present UFG high-Mn steel is attributed to the good strain-hardening ability even after yielding at high stress levels.

The yield strength and tensile strength ($\sigma_{TS}$) obtained from the stress–strain curves of the specimens were plotted as a function of minus square root of the mean grain size in Fig. 2(b) (Hall–Petch plot). For the yield strength, 0.2% offset stresses ($\sigma_{0.2}$) were taken for all specimens including those showing continuous yielding, and upper yield stresses ($\sigma_{UY}$) were also taken for the specimens that exhibited the yield-drop phenomena. Both the yield and tensile strengths held the Hall–Petch relationship,

1 $$\sigma_{y} = \sigma_{0} + kd^{{ - \frac{1}{2}}}$$

where $\sigma_{0}$ is the friction stress, and *k* is a constant. However, Fig. 2(b) clearly showed that when the mean grain sizes were smaller than 2.4 μm the yield strength deviated from extrapolation of the Hall–Petch plot for the coarse-grained specimens (dotted line) and showed higher values. The deviation of the yield strength became more significant as the mean grain size decreased, and the deviated yield strength also followed the Hall–Petch relationship. There was no obvious difference in the Hall–Petch relationship between the upper yield strength and the 0.2% offset stress for the specimens showing discontinuous yielding. The specimens with the grain sizes smaller than 2.4 μm showed a higher Hall–Petch slope (i.e., *k*, in the Hall–Petch relationship) than the specimens with the grain sizes larger than 2.4 μm. Such a deviation with a higher Hall–Petch slope has been also reported in various metals and alloys with ultrafine grain sizes^21,34,35,38^ and called extra Hall–Petch hardening^42^. It should be noted that the grain size below which the deviation occurred in the Hall–Petch relationship exactly corresponded to the critical grain size started to show the yield-drop phenomenon. This indicated that the extra Hall–Petch hardening was caused by the change in the yielding mechanism from continuous yielding to discontinuous yielding. The tensile strength did not show a deviation or extra-hardening but laid on a single straight line. The Hall–Petch relationship for the tensile strength had a similar slope to that for the yield strength of the specimens with the mean grain sizes larger than 2.4 μm. The difference between the tensile strength and the yield strength in Fig. 2(b) indicated the good strain-hardening capability maintained even in the UFG specimens of the present high-Mn steel.

### Change of dislocation density during tensile deformation

**Figure 3:**
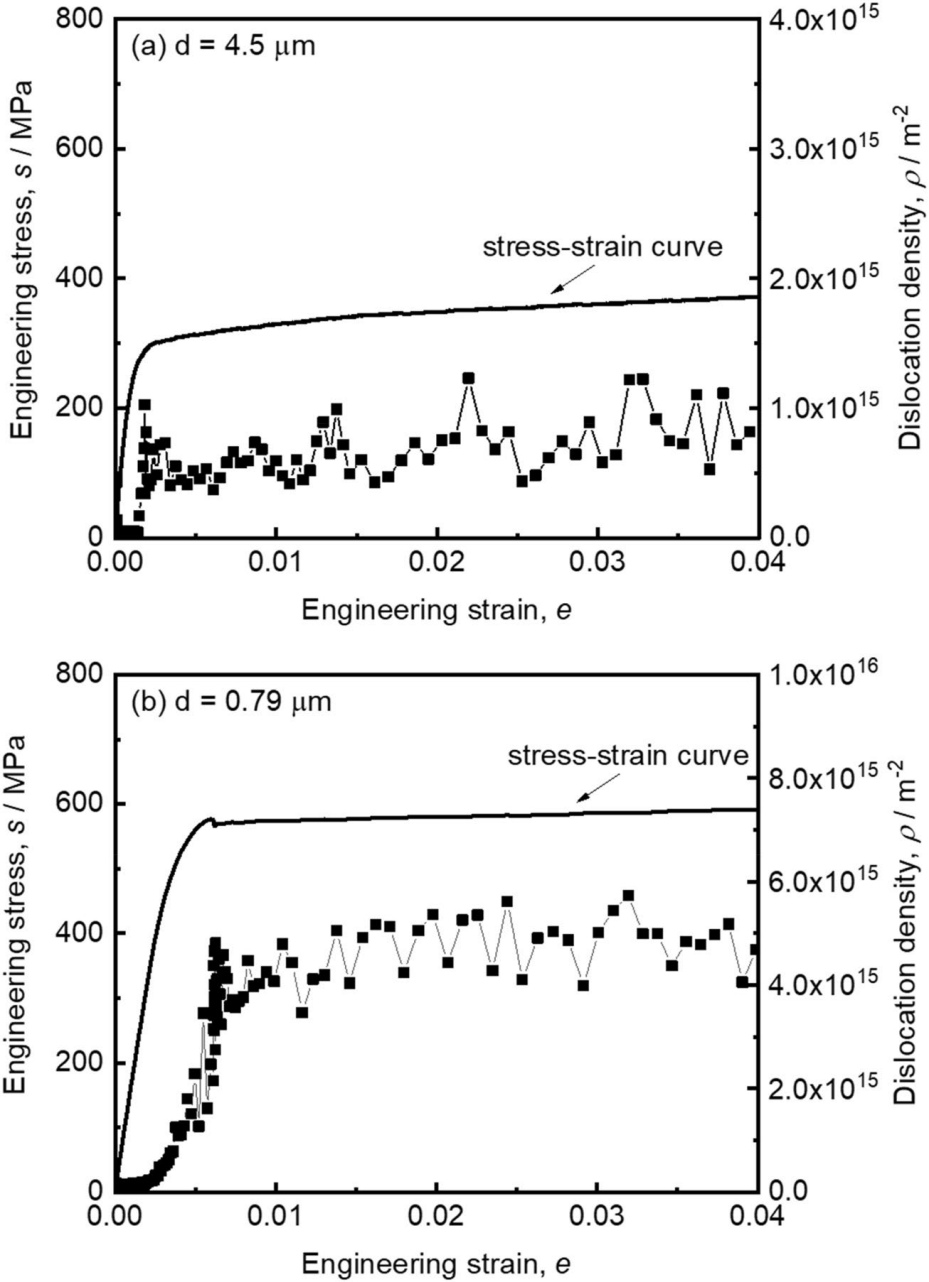
Engineering stress–strain curves and changes in dislocation densities in the small tensile strain region around macroscopic yielding in the specimens with the mean grain sizes of (**a**) 4.5 μm, and (**b**) 0.79 μm. The dislocation densities were evaluated from the results of the in-situ XRD measurements.

Changes of dislocation density in the 31Mn-3Al-3Si steel specimens were measured by the in-situ synchrotron radiation XRD measurement during tensile deformation. Figure 3(a) shows the stress–strain curve around yielding and the corresponding dislocation density obtained from Williamson-Hall method for the in-situ XRD results in the 4.5 μm grain-sized specimen. The initial dislocation density before the tensile test was 6.5 × 10^13^ m^−2^. The specimen showed normal continuous yielding, and the dislocation density started to increase before macroscopic yielding and reached to 1.0 × 10^15^ m^−2^ at the engineering strain of 0.0021 (0.21%) which corresponded to macroscopic yielding. With further increase of the engineering strain, the dislocation density increased gradually, although there were fluctuations of the dislocation densities observed sometimes, which would be within measurement error in the present experiments. Figure 3(b) represents the stress–strain curve and the dislocation density during tensile deformation of the UFG specimen with the average grain size of 0.79 μm. The initial dislocation density before the tensile test was 8.9 × 10^13^ m^−2^. In contrast to the 4.5 μm grain-sized specimen, the dislocation density of the UFG specimen did not increase so much in the elastic region, but quickly increased around the yield-point to become 4.6 × 10^15^ m^−2^ at the strain of 0.0068 corresponding to the lower yield point on the stress–strain curve. Such a quick and large increase in dislocation density in the UFG high-Mn steel implied that the deformation behavior in the UFG specimen showing discontinuous yielding was different from that in the 4.5 μm grain-sized specimen which showed typical features of FCC metals. The dislocation density in the UFG specimen in the later deformation stages after yielding (*ρ* ~ 5 × 10^15^ m^−2^) was higher than that in the 4.5 μm grain-sized specimen (*ρ* ~ 0.7–0.8 × 10^15^ m^−2^), which confirmed the good strain-hardening ability (or good strain-hardening rate) even under high stress levels in the UFG specimen described in the Sect. 2.2 above. It is considered that planner defects may affect the dislocation density calculated by in-situ XRD measurement. The influence of planer defects on dislocation density calculation is discussed in the supplementary material. In addition, it should be noted that, dislocation densities dropped rapidly after fracture in the tensile tests. Dislocation densities decreased by 54% and 68% compared with that at the ultimate tensile strength in the 4.5 μm and 0.79 μm grain-sized specimens, respectively. Such a decrease in dislocation density at fracture or unloading was also reported in pure Al^43^ and pure Cu^44^ with various grain sizes. Elastic anisotropy^45^, recovery, rearrangement and annihilation of dislocations^44^ have been considered as possible reasons for the drop of dislocation density at fracture or unloading. Anyway, the experimental evidences suggest that the dislocation density during deformation under loading is higher than that conventionally estimated by TEM measurement after unloading. It can be considered from such results that the good strain hardening in the UFG specimen is attributed to the high dislocation densities in the material.

### Evolution of deformation microstructures during tensile deformation

**Figure 4:**
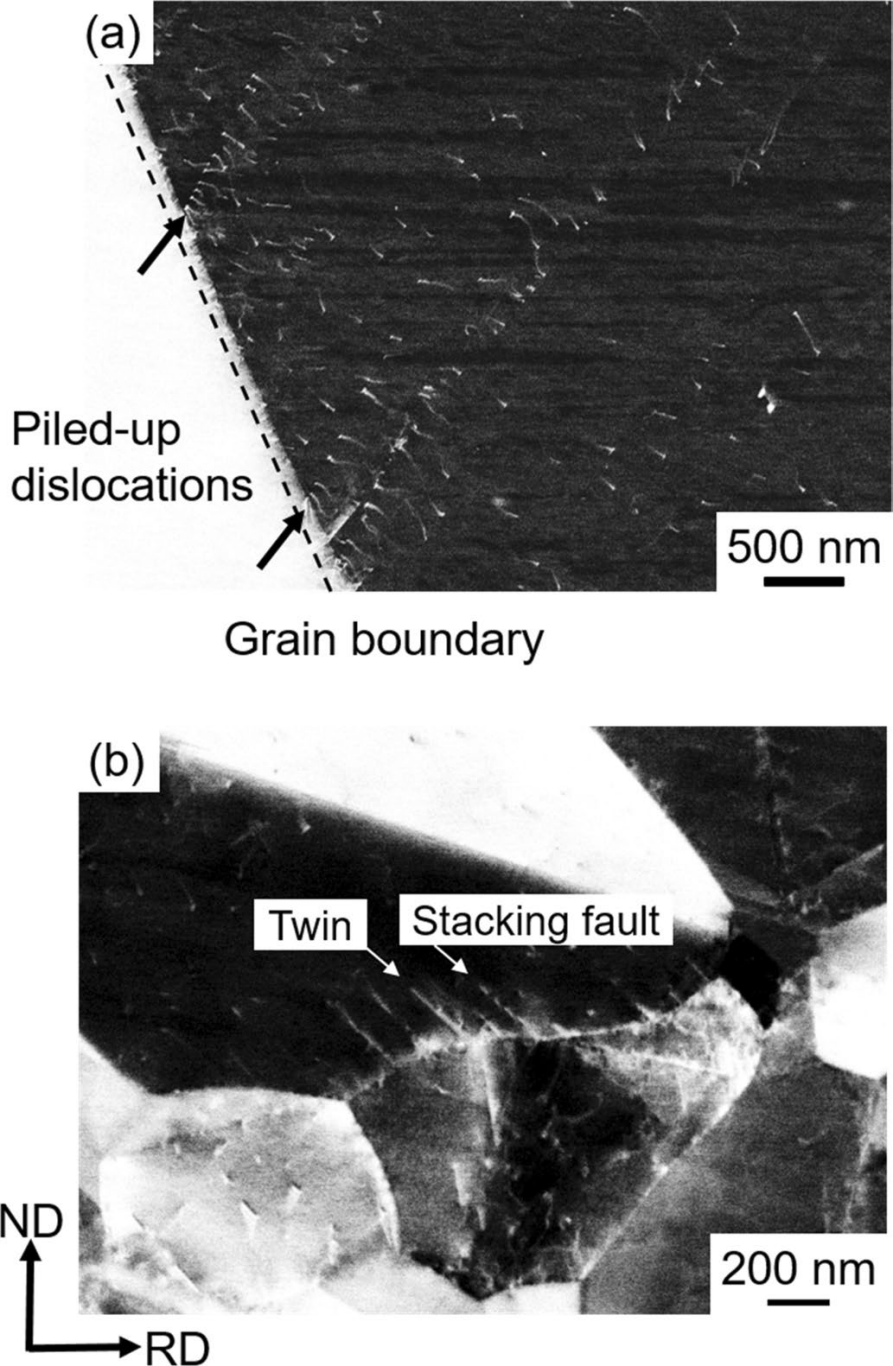
ECC images of the specimens with the mean grain sizes of (**a**) 15.4 μm and (**b**) 0.79 μm deformed to the engineering strains (*e*) of 0.007 and 0.016, respectively. The broken line in (**a**) indicates a grain boundary. The grain on the left side of the grain boundary showed a uniform white contrast, because orientation of the grain did not satisfy the Bragg condition under the present ECCI observation condition. Piled-up dislocations, stacking faults and deformation twins were indicated by arrows in the figures. (**b**) was shown in^48^ previously.

The results shown above indicated that the deformation behavior of the UFG specimen in yielding and strain-hardening stages was different from that of the specimens with the grain sizes larger than 4.5 μm. In order to understand the deformation mechanism, deformed microstructures in the tensile-deformed specimens were carefully observed by the ECCI technique^46,47^. Figure 4(a) shows an ECC image of the 15.4 μm grain-sized specimen after tensile deformation to an engineering strain of 0.007 (0.7%), corresponding to a deformation stage just after yielding. The broken line in Fig. 4(a) indicates a grain boundary. Typical dislocation pile-ups at the grain boundary was observed, which corresponded to a conventional continuous yielding in the coarse-grained specimen. Figure 4(b) shows an ECC image of the UFG specimen (*d* = 0.79 μm) after tensile deformation to a strain of 0.016 (1.6%), corresponding to a deformation stage just after the yield-drop^48^. A small number of dislocations were observed inside grains, but they did not pile up at grain boundaries. On the other hand, deformation twins and stacking faults seemed to nucleate from grain boundaries in the UFG specimen. The deformation twins and stacking faults were distinguished from their thickness in higher magnification images.

**Figure 5:**
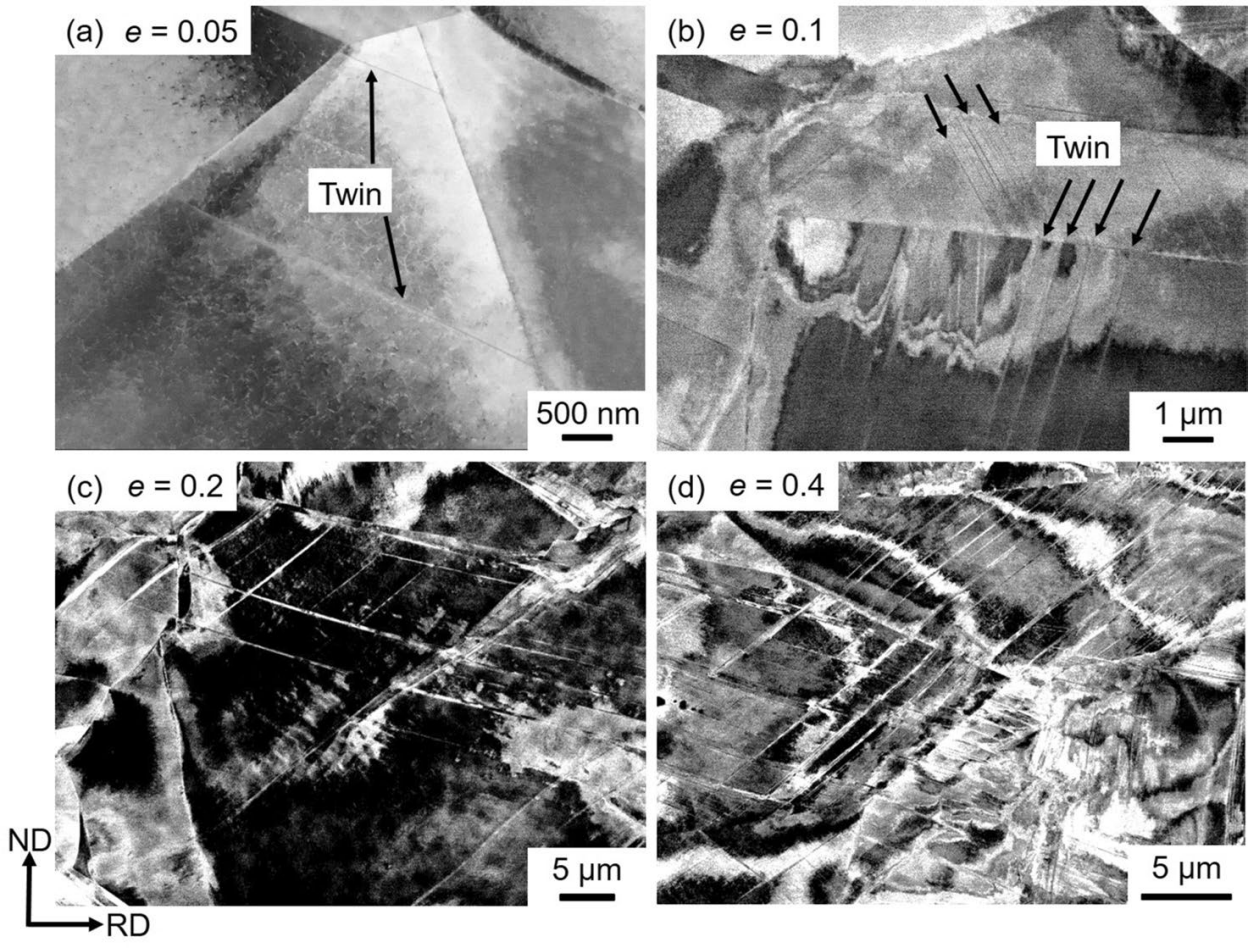
ECC images of the specimens with the mean grain sizes of 15.4 μm tensile-deformed to engineering strains of (**a**) *e* = 0.05, (**b**) 0.1, (**c**) 0.2 and (**d**) 0.4.

**Figure 6:**
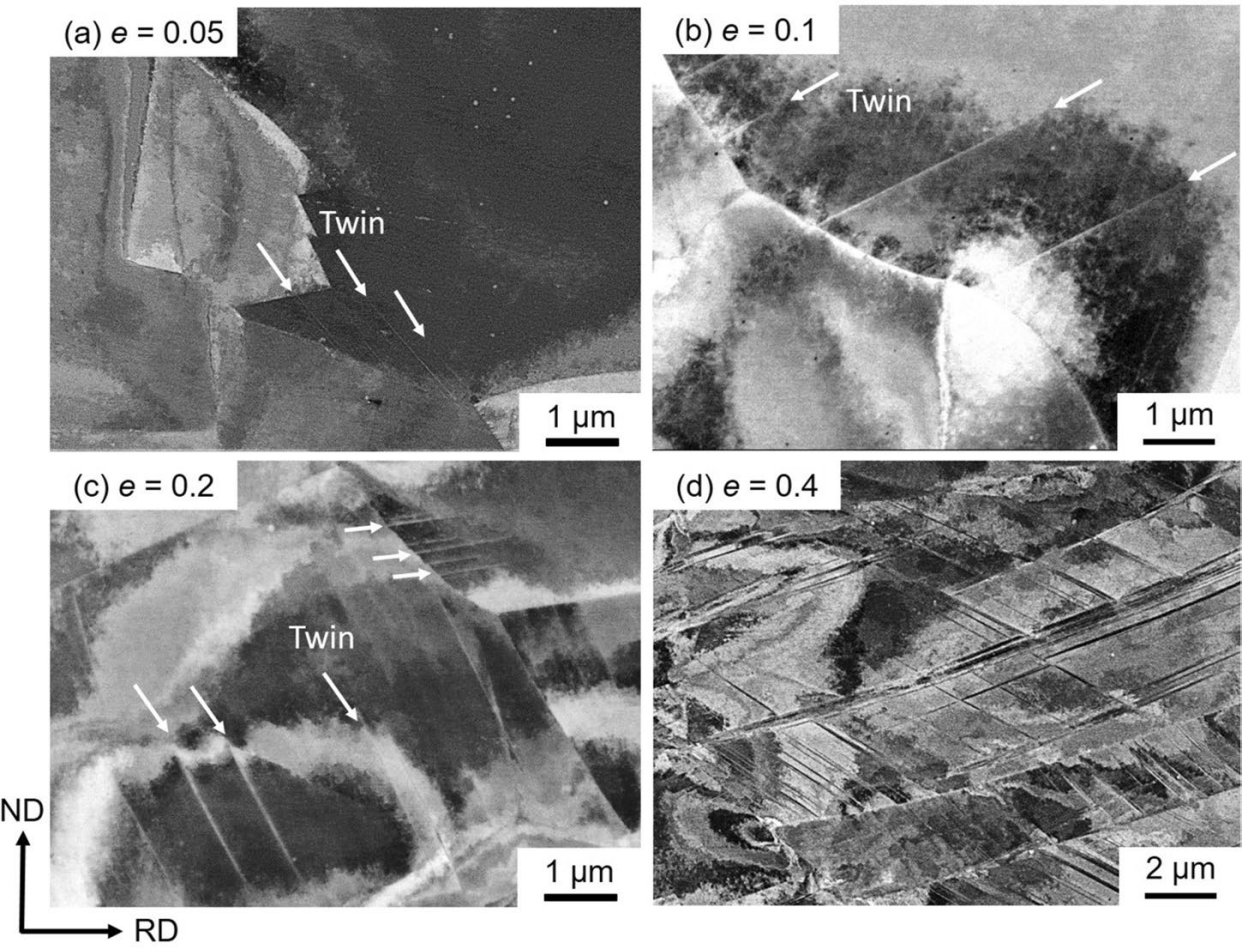
ECC images of the specimens with the mean grain sizes of 4.5 μm tensile-deformed to engineering strains of (**a**) *e* = 0.05, (**b**) 0.1, (**c**) 0.2 and (**d**) 0.4.

**Figure 7:**
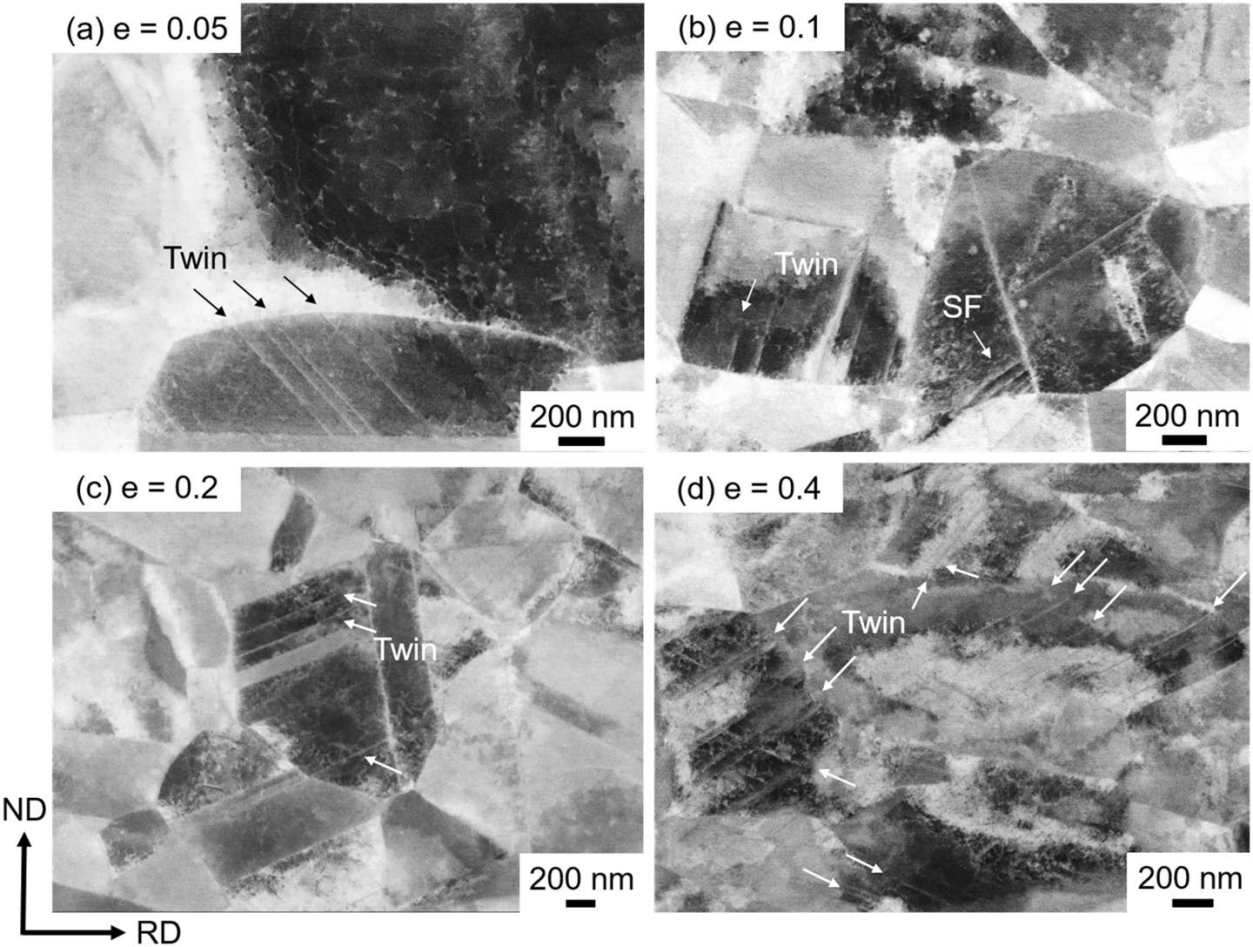
ECC images of the ultrafine-grained (UFG) specimens with the mean grain sizes of 0.79 μm tensile-deformed to engineering strains of (**a**) *e* = 0.05, (**b**) 0.1, (**c**) 0.2 and (**d**) 0.4.

Microstructure evolution in the specimens with the mean grain sizes of 15.4 μm, 4.5 μm, and 0.79 μm was characterized by ECCI after tensile deformation to different tensile engineering strains of 0.05, 0.1, 0.2 and 0.4, and the results are shown in Figs. 5, 6 and 7, respectively. Microstructure of the 15.4 μm grain-sized specimen deformed to a strain of 0.05 consisted of dislocations and a few numbers of very thin deformation twins (indicated by arrows) of a single twinning system, as shown in Fig. 5(a). At a strain of 0.1 (Fig. 5b), the number of deformation twins increased. The matrix showed dislocation substructures, and planer arrays of dislocations were often recognized. The number of deformation twins furthermore increased with increasing the tensile strain to 0.2 (Fig. 5c) and 0.4 (Fig. 5d). Dislocation substructures in the matrix were difficult to be clearly recognized by ECCI in the specimens deformed to a strain of 0.4, possibly due to the increase of the dislocation density.

Evolution of deformation microstructures in the specimen with the average grain size of 4.5 μm was similar to that in the specimen with the grain size of 15.4 μm, as shown in Fig. 6(a–d). Compared with the 15.4 μm grain-sized specimen (Fig. 5), however, the thickness and number of deformation twins at equivalent strains somehow decreased in the 4.5 μm grain-sized specimen (Fig. 6).

**Figure 8:**
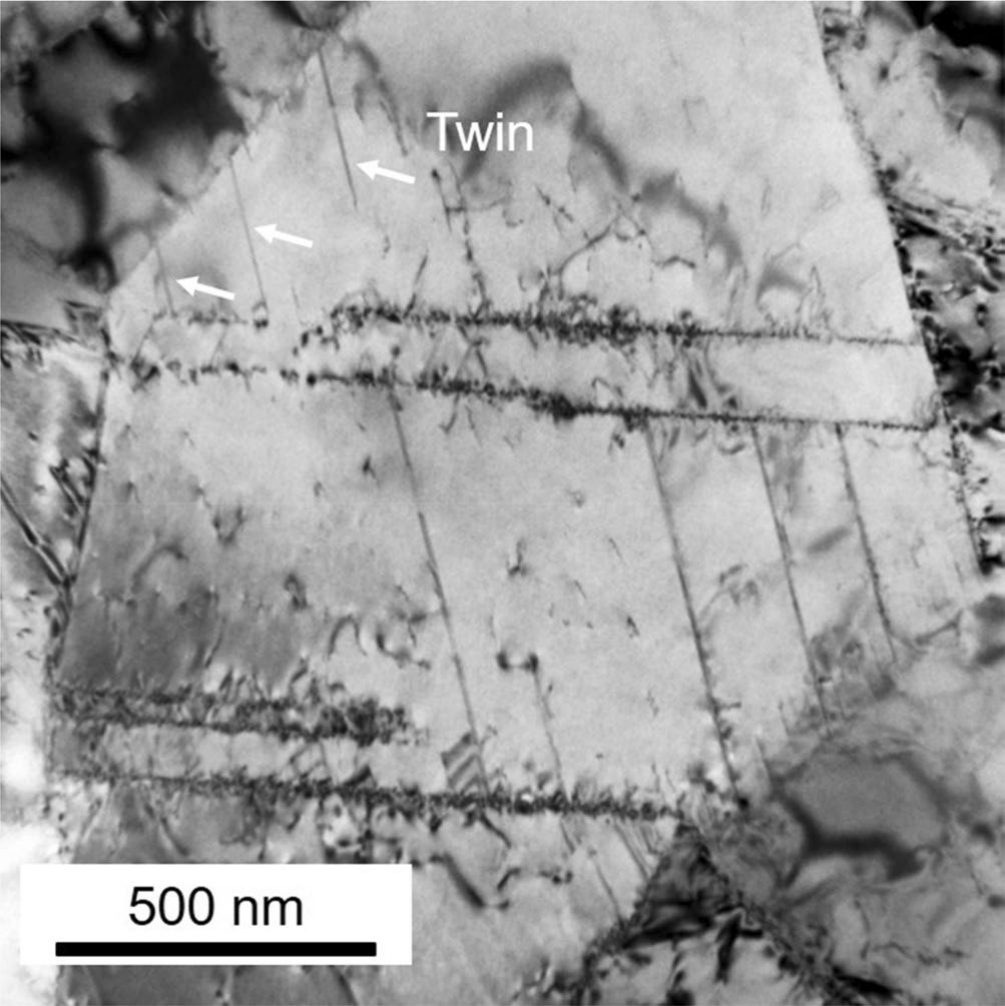
Bright field TEM image of the ultrafine-grained (UFG) specimen with the mean grain size of 0.79 μm tensile-deformed to an engineering strain of *e* = 0.062.

It should be noted that, in the UFG specimen having the grain size of 0.79 μm, higher density of dislocations and larger number of thin deformation twins were observed at an engineering strain of 0.05 (Fig. 7a) than in the 4.5 μm grain-sized specimen at the same strain level (Fig. 6a). As the tensile strain increased to 0.1, stacking faults were often observed in addition to dislocations and thin deformation twins (Fig. 7b). The deformation twins and stacking faults seemed to nucleate from grain boundaries, as were pointed out by arrows in Fig. 7, although most deformation twins observed in the specimens with the grain sizes of 15.4 μm and 4.5 μm were also connected their ends to grain boundaries (Figs. 5 and 6). In our parallel study, deformed microstructures of the present high-Mn steel were carefully examined by transmission electron microscope (TEM)^49^. Figure 8 showed a representative bright field TEM image of the UFG specimen with the grain size of 0.79 μm after a tensile deformation to a strain of 0.062. It was clearly seen that several deformation twins (indicated by white arrows) generated from grain boundaries, which was consistent with the result of the ECC image shown in Fig. 7(b). The detailed characterization of microstructures is discussed in our another paper^49^. At strains of 0.2 and 0.4 (Fig. 7c and d), the number of deformation twins increased. Since deformation twins generated in the UFG specimens were very thin, it was difficult to evaluate thickness of deformation twins precisely only by ECCI observation. Thus, TEM observation was conducted for estimating the thickness of deformation twins in the present study. The average thicknesses of deformation twins were 204 nm, 43 nm, 31 nm in the 15.4 μm, 4.5 μm and 0.79 μm grain-sized specimens, respectively, at the engineering tensile strain of 0.4. The thickness of twins decreased with decreasing the matrix grain size, and it was quite thin in the UFG specimen. Several studies have been investigated the effect of grain size on deformation twinning, especially thickness of deformation twins in the UFG and nanocrystalline Cu–Zn alloys with low stacking fault energies^50,51^. Although the UFG and nanocrystalline structures in these papers were fabricated by severe plastic deformation, it was found that deformation twin thickness decreased with decreasing the grain size. The result about the twin thickness obtained in the present study is consistent with the above mentioned previous study.

**Figure 9:**
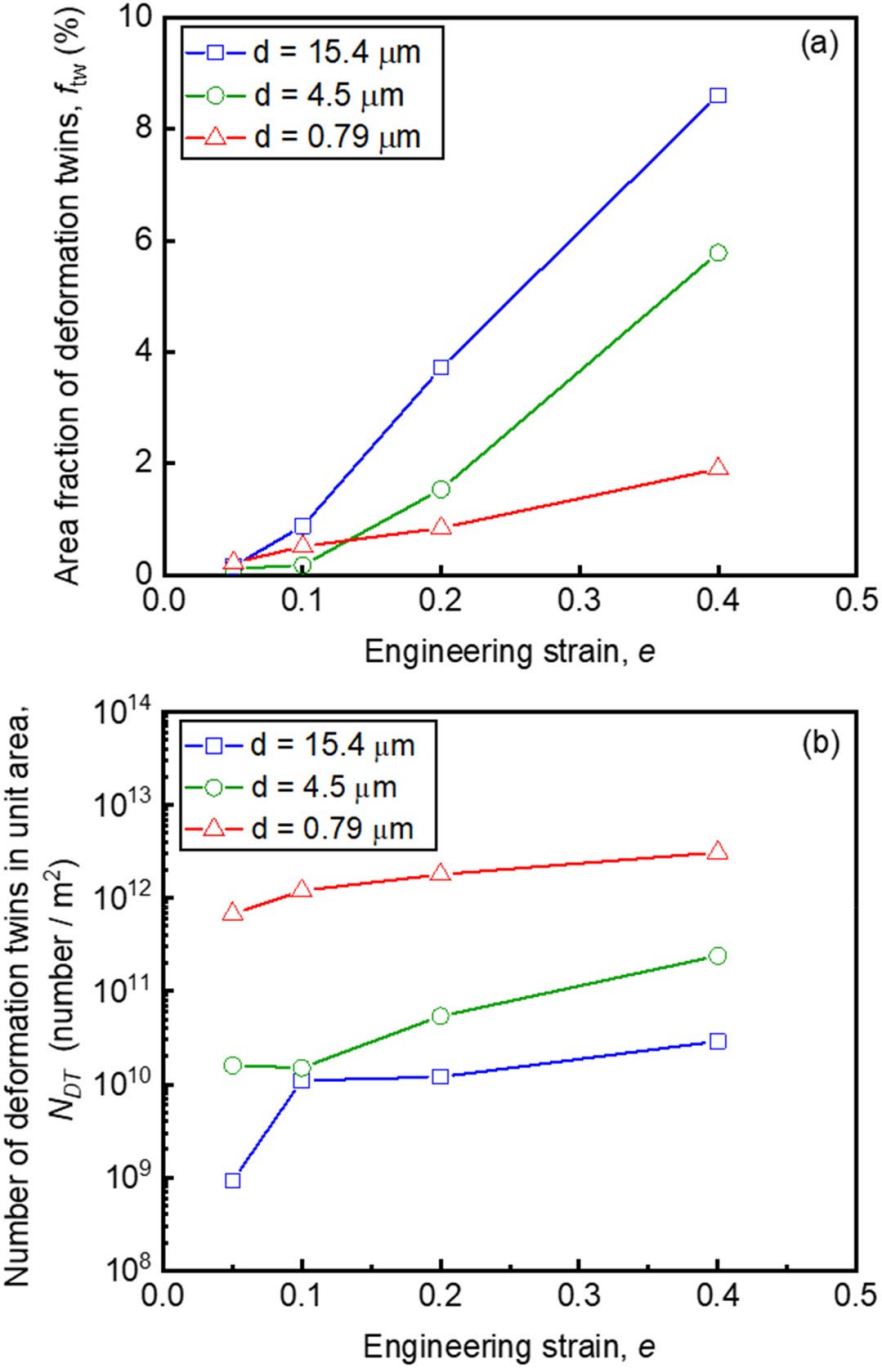
(**a**) Area fractions of deformation twins (*f*_tw_) and (**b**) number of deformation twins in unit area (*N*_*DT*_) obtained from the ECC images of the tensile-deformed specimens having different mean grain sizes of 15.4 μm, 4.5 μm and 0.79 μm. Plotted as a function of the engineering strain.

Deformed microstructures of the specimens with different grain sizes were carefully observed by means of ECCI. The quantitative result of the area fractions of deformation twins (*f*_tw_) in the specimens having different grain sizes are plotted as a function of the tensile engineering strain in Fig. 9(a). At a strain of 0.05, *f*_tw_ was very small in all the specimens with different mean grain sizes. When the tensile strain increased to 0.1, *f*_tw_ was still small but increased to 0.89%, 0.18% and 0.52% in the 15.4 μm, 4.5 μm and 0.79 μm grain-sized specimens, respectively. Here we would like to pay an important attention to the fact that *f*_tw_ of the UFG specimen was obviously larger than that of the 4.5 μm grain-sized specimen. It has been reported that deformation twinning is suppressed with decreasing the matrix grain size in FCC metals and alloys^3,52,53^. The present result that *f*_tw_ of the 15.4 μm grain-sized specimen was always larger than those of the 4.5 μm grain-sized and UFG specimens at all tensile strains matched with such a well-known tendency. Above tensile strain of 0.2, *f*_tw_ of the UFG specimen became smaller than that of the 4.5 μm grain-sized specimen. Figure 9(b) showed the number of deformation twins in unit area (*N*_*DT*_, number density) plotted as a function of the tensile engineering strain. Number of deformation twins increased with increasing the engineering strain in all the specimens. It should be noted that the UFG specimen showed significantly larger numbers of deformation twins than those in the 4.5 μm and 15.4 μm grain-sized specimens at the same strain levels. The higher number density of twins in the UFG specimen could be also confirmed in ECC images (Fig. 7). Considering the area fraction and the number density of deformation twins shown in Fig. 9, it can be concluded that deformation twinning was rather enhanced in the UFG specimen especially at early stage of deformation, contrary to the conventionally known grain size dependence of deformation twinning in FCC metals and alloys. Such results suggested that the deformation behavior in the 0.79 μm grain-sized specimen was significantly changed by the ultra grain refinement, corresponding to the change of yielding behavior from continuous yielding into discontinuous yielding.

## Discussion

The yield-drop phenomenon typically observed in pure iron and low carbon steels with BCC structure has been well recognized and explained by the Cottrell-Bilby theory^32^. Interstitial solute atoms (i.e., carbon and nitrogen) in commercial pure iron and low carbon steels interact with dislocations to form Cottrell atmosphere. Dislocations are locked by interstitial atoms under the Cottrell atmosphere, so that the external stress necessary to move dislocations increases. When the dislocations are unlocked from the atmosphere at a certain level of external stress, large scale plastic deformation suddenly starts in a localized manner (in the form of Lüders band), leading to the yield-drop observed on the stress–strain curve.

In the present study, the 31Mn-3Al-3Si steel having average grain sizes larger than 2.4 μm showed continuous yielding, which was typical in almost all coarse-grained FCC metals and alloys. However, the 31Mn-3Al-3Si specimens with grain sizes of 1.5 μm or smaller exhibited discontinuous yielding characterized by obvious yield-drop and localized deformation (Lüders band). Before the present study, such yield-drop phenomena have also been found in other kinds of fully recrystallized UFG metallic materials^29,34,35,37–40,42^, nevertheless coarse-grained specimens of the same materials showed continuous yielding without yield-drop. Accordingly, it can be said that the yield-drop phenomenon is a unique property universally observed in recrystallized UFG polycrystalline materials regardless of their crystal structures and chemical compositions. In the present FCC high-Mn steel, there were no interstitial solute atoms that could interact with dislocations to form the Cottrell atmosphere. This indicates that the reason for the yield-drop in the present UFG high-Mn steel is completely different from that for the ferritic iron and steels explained by dislocation locking by interstitials and unlocking.

The yield-drop phenomenon can be generally understood in terms of the lack of mobile dislocations in the materials. The situation in the conventional pure iron and low carbon steels where dislocations are locked by interstitial atoms can be also understood in the same context since the locked dislocations are immobile till unlocking. The in-situ synchrotron radiation XRD analysis in Fig. 3 revealed that the dislocation density in the 4.5 μm grain-sized specimen started to increase before macroscopic yielding and reached to 1.0 × 10^15^ m^−2^ at the engineering strain of 0.0021 (0.21%) which corresponded to macroscopic yielding, while the dislocation density in the UFG specimen (*d* = 0.79 μm) did not increase so much in the elastic region, but suddenly increased around the yield point to become 4.8 × 10^15^ m^−2^ at the strain of 0.0071 corresponding to the lower yield point on the stress–strain curve. The increment of dislocation density around macroscopic yielding was much more significant in the UFG specimen than that in the 4.5 μm grain-sized specimen. The result seems to suggest a lack of mobile dislocations at the initial stage of the UFG specimen. Since the present specimens were all fully recrystallized, dislocation densities before tensile tests were certainly low. However, the dislocation densities were not so different between the UFG specimen and the coarse-grained specimen. Here, we would like to pay attention to different volumes of grains^54^. Assuming a simple cubic shape of grains, the average numbers of dislocations in the 0.79 μm and 4.5 μm grain-sized specimens with initial dislocation densities of 8.9 × 10^13^ m^−2^ and 6.5 × 10^13^ m^−2^ (measured values by synchrotron XRD) are simply calculated as 55 and 1316, respectively. The number of dislocations per grain in the UFG specimen is very limited compared to that in the 4.5 μm grain-sized specimen. The slip length of dislocations (mean free path) in each ultrafine grain is limited, so that large plastic strain cannot be gained even when the limited numbers of pre-existing dislocations glide. Additionally, the small volume of the ultrafine grain inhibits multiplication of dislocations within the grain, as was predicted by Ohashi et al.^55^. Consequently, initiating macroscopic yielding becomes difficult in UFG polycrystalline materials with recrystallized structures owing to the scarcity of free dislocations in each grain. For initiating macroscopic plastic deformation in such recrystallized UFG metals and alloys, it is necessary to nucleate lattice defects that can bring about plastic deformation, such as dislocations, deformation twins, etc., which has been argued in our recent paper on a concept of “*plaston*” emphasizing the importance of the nucleation of deformation modes for realizing both high strength and large plasticity in UFG materials^48,56^. Once a local stress reaches the critical value necessary for nucleating a lattice defect bringing about plastic deformation, macroscopic yielding of the material starts under the tensile deformation at a constant displacement speed. It should be noted that the transition from continuous yielding to discontinuous yielding occurred between 2.4 μm and 1.5 μm. The authors do not have in-situ synchrotron diffraction data and dislocation densities before tensile test for the 2.4 μm and 1.5 μm grain-sized specimens. However, assuming the initial dislocation density same as that in the 0.79 μm grain-sized specimen (8.9 × 10^13^ m^−2^), the number of dislocations existing in each grain of the 2.4 μm or 1.5 μm grain-sized specimens is calculated to be 513 or 200, respectively. We do not know the critical number of dislocations in each grain for inducing discontinuous yielding, the simple calculation suggests it would be in an order of 10–100.

The extra-hardening in the ultrafine grain-size region in the Hall–Petch plot shown in Fig. 2(b), which exactly corresponded to the transition of yielding behavior into discontinuous yielding accompanying with yield-drop. This result supports the above-mentioned consideration that a higher stress is necessary to initiate macroscopic yielding in the UFG specimens. That is, the extra Hall–Petch hardening is considered to be also originated from the scarcity of free dislocations and dislocation sources in each grain of the recrystallized UFG materials, which could be understand as a kind of source hardening. Furthermore, local strain distributions in the tensile specimens during tensile tests were analyzed by the DIC technique and the result shown in supplementary Figure S1 clearly indicated that the initiation of plastic deformation did not happen homogeneously in the material but occurred in a localized manner, i.e., in the form of Lüders banding. Such localized manner of deformation initiation is another reason for the yield-drop.

It would be reasonable to consider that such lattice defects are nucleated from grain boundaries in the UFG materials where the density of grain boundaries rises very much. In fact, a number of thin deformation twins and stacking faults formed from grain boundaries in the UFG specimen around yielding (Figs. 4b, 7a and 8). By now several nucleation models for deformation twins in FCC metals^9,57–60^ have been proposed. All those nucleation mechanisms are explained by dislocation interactions from geometric and crystallographic points of view, which can explain the conventionally recognized grain size dependence in coarse-grained FCC metals and alloys^55^. Namely, the larger the grain size, the higher the possibility of dislocation interactions within grains, leading to more frequent formation of deformation twins. In this context, when the grain size becomes very small, the number of dislocations in each recrystallized grain becomes small (as discussed above for explaining the yield-drop), so that the chance of dislocation interaction decreases and in-grain nucleation of deformation twins becomes difficult. On this basis, it has been conventionally considered that the grain refinement inhibits deformation twinning in FCC metals and alloys. In fact, it has been reported that deformation twinning was suppressed by grain refinement in 31Mn-3Al-3Si and 22Mn-0.6C TWIP steels^3,25,26^. It should be noted, however, the minimum average grain sizes of the TWIP steels examined in the previous studies were 2–3 μm. In the present study, a large number of very thin deformation twins were observed in the UFG 31Mn-3Al-3Si steel specimen with the average grain size of 0.79 μm after tensile deformation (Figs. 4a and 7). The number density of deformation twins in the UFG specimen was much higher than that in the 4.5 μm grain-sized specimen at a tensile strain of 0.1 (Fig. 8b). As expected above, deformation twins and stacking faults mostly nucleated from grain boundaries in the UFG specimens. This was probably because the stress level in the UFG specimens increased due to the scarcity of free dislocations in each grain to become high enough to nucleate twins, and the density of nucleation sites for twins (i.e., grain boundaries) increased by the grain refinement. Nucleation of deformation twins, stacking faults and dislocations are probably not competitive in the UFG specimen, since both deformation twins and stacking faults are observed in Fig. 4(b) and also both deformation twins and dislocations are recognized in Fig. 7(a). This consideration would be reasonable because deformation twin (as well as stacking fault) in FCC metals and alloys is formed by glide of leading Shockley partial dislocation. Under the situation without enough number of free dislocations and dislocation sources (described above), once a local stress reaches the critical value, any lattice defects would be nucleated from grain boundaries^48^.

Accordingly, such twinning behavior found in the current UFG high-Mn steel is quite unique. The current results revealed that initial deformation mechanisms switch from simple glide and multiplication of pre-existing dislocations in conventionally coarse-grained material into nucleation of deformation twins, stacking faults and dislocations from grain boundaries in the UFG high-Mn steel, together with the transition of yielding behavior from continuous to discontinuous manners. The unique transition of yielding behavior from continuous to discontinuous manner by grain refinement could be understood on the basis of limited number of free dislocations in each ultrafine grain. The thinner twins in the finer matrix grain size could result from such a transition of twinning mechanism in the fine grained (*d* = 1.5 μm) and UFG specimens. Furthermore, the formation of thinner deformation twins might be the key to the enhanced strain-hardening and resultant high strength and large tensile ductility of the UFG specimens. The unique transition of deformation and twinning behavior in the present high-Mn TWIP steel in the sub-micrometer grain size regime is a new finding which has not been reported by now. In our following papers, we clarify the shift of deformation mechanism with the grain refinement in more detail by advanced TEM observations^49^, and show the nucleation mechanism of deformation twins from particular grain boundaries by the aid of postmodern and in-situ TEM techniques^61^.

## Conclusions

The change of mechanical properties and the nature of unique yielding behavior of the UFG 31Mn-3Al-3Si high-Mn TWIP steel were investigated. The specimens having the grain sizes larger than 2.4 μm showed continuous yielding, while discontinuous yielding accompanying with yield-drop was observed when the mean grain size was 1.5 μm or smaller. The discontinuous yielding in the UFG specimens could be explained by the scarcity of free dislocations in each UFG grain, which was experimentally supported by the quick increase of dislocation density around yielding in the 0.79 μm grain-sized specimen measured by the in-situ synchrotron radiation X-ray diffraction. The scarcity of free dislocations increases the critical stress for initiating macroscopic yielding, which can also interpret the extra Hall–Petch hardening in the UFG specimens. The ECCI observation showed nucleation of deformation twins and stacking faults from grain boundaries in the UFG specimen around yielding. The number density of deformation twins in the 0.79 μm grain-sized specimen was much higher than that in the 4.5 μm grain-sized specimen, even though it has been conventionally understood that deformation twinning is suppressed by the grain refinement. The unusual enhancement of deformation twinning in the UFG specimen could be also explained by the scarcity of free dislocations in each recrystallized UFG grain. The frequent formation of nano-twins was also considered as one of the important reasons for the enhanced strain-hardening ability in the UFG specimens which led to both high strength and large tensile ductility. The obtained results indicated a unique transition of deformation mechanisms in the high-Mn TWIP steel depending on the grain size.

## Methods

### Materials

A 31Mn-3Al-3Si steel (Mn: 31.0, Al: 3.0, Si: 3.0, C: 0.005, N: 0.004, S: 0.012, Fe: bal. (mass %)) having an austenite single-phase structure was used in the present study. The high-Mn steel is a typical C-free TWIP steel having relatively low strength but very good ductility. The stacking fault energy of this material is reported to be less than 40 mJ m^−2^ according to a thermodynamic model^62^. As-received material was a hot-forged sheet with a thickness of 12 mm. Multi-pass cold-rolling with 92% total reduction in thickness (corresponding equivalent strain *ε* = 2.87) was conducted to make a sheet 1 mm thick. A two-high rolling mill with a roll diameter of 250 mm was used for the cold-rolling under a lubricated condition at room temperature. The cold-rolled material was annealed at different temperatures ranging from 650 °C to 1100 °C for different periods from 300 to 3600 s, and then followed by water cooling to obtain fully recrystallized specimens having various mean grain sizes including ultrafine grain sizes.

### Microstructural observations

Microstructures of the specimens after the cold-rolling and subsequent annealing process were characterized by the use of a field-emission scanning electron microscope (FE-SEM) equipped with a back scattered electron (BSE) detector and an electron back-scattering diffraction (EBSD) system^29^. The samples for the SEM-BSE and SEM-EBSD observations were mechanically polished with emery papers and then electro-polished in a solution of 90 vol. % ethanol + 10 vol. % perchloric acid at room temperature with a voltage of 30 V for 30 s to obtain mirror surfaces. All microstructural observations were carried out on longitudinal sections perpendicular to the transverse direction (TD) of the sheets. Mean grain sizes were measured using the line intercept method on grain boundary maps obtained by EBSD, where all high angle grain boundaries including annealing twin boundaries were counted.

### Tensile tests

Uniaxial tensile tests at room temperature were carried out to evaluate mechanical properties of the recrystallized specimens with various mean grain sizes. Sheet-type tensile specimens with a gage length of 10 mm and width of 5 mm, which was the 1/5 miniaturized size of the JIS-5 specimen, were cut from the 1 mm thick annealed sheets by an electrical discharge machine. Tensile tests were conducted at room temperature by the use of an Instron-type tensile test machine (Shimadzu, AG-100kN Xplus). The tensile direction was parallel to the rolling direction (RD) of the sheets. The crosshead speed in the tensile test was 0.5 mm min^−1^, corresponding to an initial strain rate of 8.3 × 10^–4^ s^−1^. An extensometer was attached to the tensile specimens in order to measure the displacement in tensile deformation precisely. Digital image correlation (DIC) method^63,64^ was used to measure local strain distributions in the tensile test specimen during deformation. Broad surfaces of the tensile specimens were painted in white, and then black paint was sprayed to make random black speckle patterns as markers to track displacements of different local positions during the tensile test. The area of gage part (length of 10 mm and width of 5 mm) and shoulder parts of tensile specimens were captured at a rate of 5 frames per second by a charge-coupled device (CCD) camera with a resolution of 2432 × 2054 pixels during the tensile deformation continuously. The CCD images of the gage parts of the tensile test specimens were observed from the nominal direction (ND) of the sheet specimens and recorded. Local strains at different positions on the broad surfaces of the specimens were calculated from the captured images by the use of a commercial digital image correlation software, Vic-2D.

### Characterization of deformed microstructure

In order to study the evolution of deformation microstructures with a progress of deformation, interrupted tensile tests were also conducted. Tensile specimens having the same dimensions described above were tensile-deformed to various strains before fracture and then deformation microstructures were quantitatively characterized. The electron channeling contrast imaging (ECCI) technique^65^ in SEM was used to characterize deformation substructures, such as deformation twins and dislocations, in the specimens deformed to various tensile strains, especially around macroscopic yielding. ECCI observations were conducted at center thickness regions on the sections perpendicular to the transverse direction (TD) of the sheet-type specimens in FE-SEM. Surfaces of the specimens for the ECCI observations were mechanically polished by emery paper and diamond paste with a particle size of 1 μm. Then electro-polishing was conducted for removing deformed layers formed by mechanical polishing. Finally, the specimen surfaces were polished by colloidal silica with 0.02 μm size for obtaining mirror surfaces. ECCI observation was performed in a SEM equipped with a back scattered electron (BSE) detector at an accelerating voltage of 25 kV, a probe current of 12 mA and a working distance in a range from 2.5 mm to 4 mm. By using those many ECC images at specific strains, the area fractions of deformation twins (*f*_tw_) and the number of deformation twins in unit area (*N*_*DT*_) in the specimens having different grain sizes were quantitatively estimated. On ECC images, deformation twins were colored in red, and the area fractions of twins were evaluated using an image software (GIMP-GNN Image Manipulation Program, V.2.8.18, https://www.gimp.org/).

### In-situ X-ray diffraction (XRD) analysis

**Figure 10:**
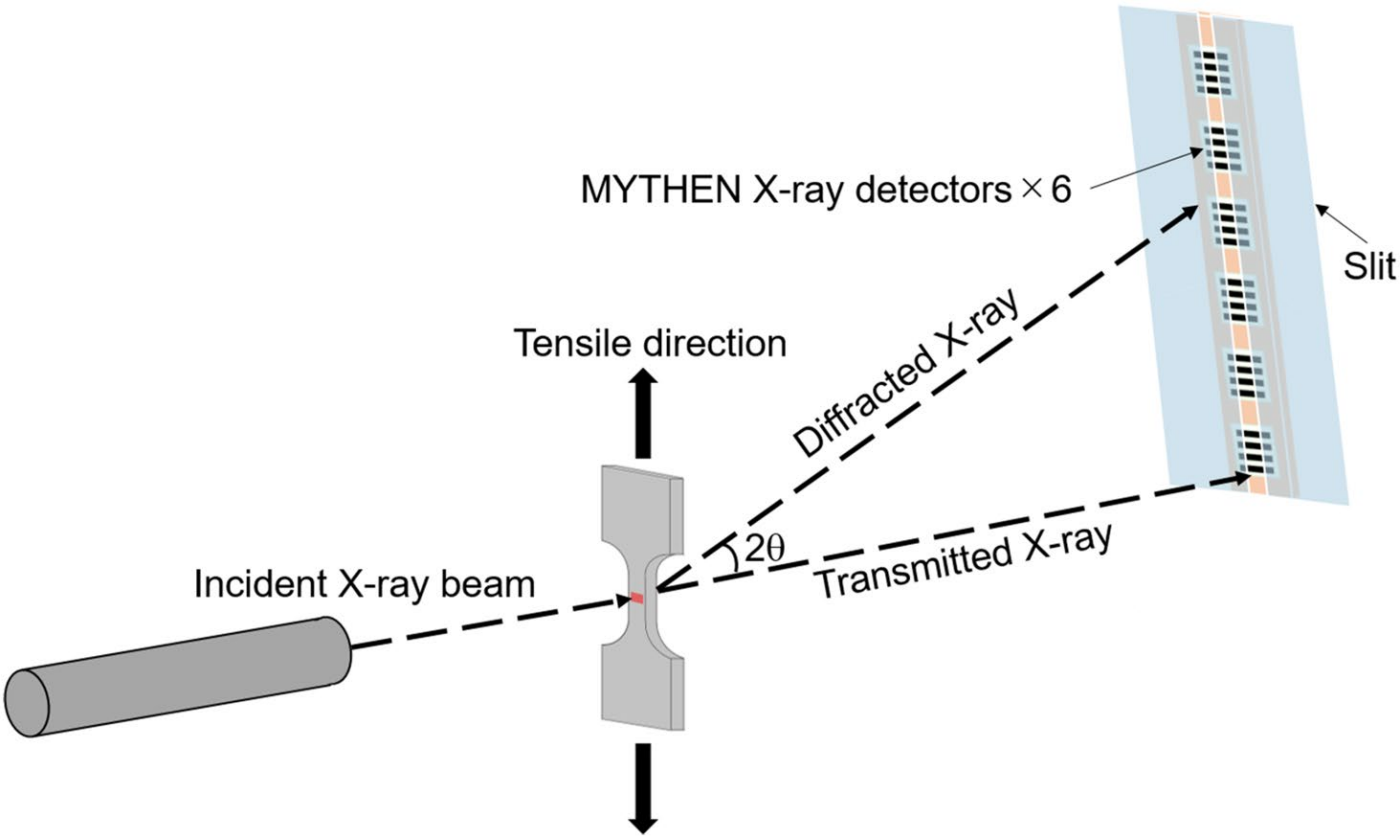
A schematic illustration showing the in-situ synchrotron XRD measurement system^66^.

In order to know changes in dislocation density in the specimens during the tensile deformation, in-situ X-ray diffraction was conducted at the beam line BL46XU of SPring-8 in Harima, Japan. The tensile tests were performed at room temperature and an initial strain rate of 8.3 × 10^–4^ s^−1^. The gage part of the tensile test specimens used for the in-situ X-ray diffraction measurement were 10 mm in length, 5 mm in width and 0.5 mm in thickness. The energy of X-ray we used was 30 keV, corresponding to a wavelength of *λ* = 0.0413 nm. The incident beam having a size of 0.5 mm and 0.3 mm in the horizontal and vertical directions was irradiated at the center of the gage part in the tensile specimen. Figure 10 schematically shows the in-situ XRD measurement system during the tensile test^66^. The incident X-ray beam was irradiated perpendicular to the tensile test specimen, and the profiles of X-ray diffraction were detected by one-dimensional detector. The one dimensional detector was consisted of six MYTHEN detectors (a one-dimensional microstrip detector made by DECTRIS) arranged in a line. The length of the sensor was about 400 mm and the width of array was 0.05 mm. The distance between the specimen and the detector was 733.96 mm, so that the range and step of diffraction angle of X-ray diffraction profile we measured was about 30 degrees and 0.004 degrees, respectively. The position of the detector was adjusted by the detector arm of the multi-axis diffractometer so that the profiles of X-ray diffraction in the range from 9.8° to 40.3° could be measured. The measurement time for each X-ray diffraction profile was 0.2 s^67^. Obtained diffraction peaks were analyzed using the pseudo-Voigt function^68^ and values of the full width at half maximum (FWHM) were obtained. The obtained data were analyzed by the Williamson-Hall method^69^ to evaluate dislocation densities *ρ*:

2 $$\frac{\Delta 2\theta \cos \theta }{\lambda } = \frac{0.9}{D} + \frac{2 \varepsilon \sin \theta }{\lambda }$$

3 $$\rho = \frac{{k \varepsilon^{2} }}{{b^{2} }}$$

where *θ* is the diffraction angle, Δ2*θ* is the full width at half maximum (FWHM) of the diffraction peak, *λ* is the wavelength of the incident X-ray beam, *ε* is the heterogeneous strain, *D* is the crystallite size, and *b* is the magnitude of Burgers vector (0.2556 nm for the present high-Mn steel). *k* is a constant related to the crystal structure, which is 16.1 for the FCC structure^70^. *ε* and *D* are evaluated from the slope and intercept of the obtained line in the Williamson-Hall plot according to the Eq. (2).

## Supplementary Information

Supplementary Information.
