# Supplementary material for: Unique transition of yielding mechanism and unexpected activation of deformation twinning in ultrafine grained Fe-31Mn-3Al-3Si alloy
Source: Sci Rep. 2021 Aug 5;11:15870. doi: 10.1038/s41598-021-94800-6 (PMC8342466; doi:10.1038/s41598-021-94800-6)
Supplement: Supplementary file 1 — Supplementary Information. [file 41598_2021_94800_MOESM1_ESM.pdf]

Supplementary Materials for

Unique transition of yielding mechanism and unexpected activation of deformation twinning in ultrafine grained Fe-31Mn-3Al-3Si alloy

Yu Bai [a,b,c*], Hiroki Kitamura [b], Si Gao [b], Yanzhong Tian [d], Nokeun Park [c,e],

Myeong-heom Park [b,c], Hiroki Adachi [f], Akinobu Shibata [b,c,g], Masugu Sato [h],

Mitsuhiro Murayama [i,j], Nobuhiro Tsuji [b,c*]

[a] School of Materials Science and Engineering, Dalian University of Technology, No.2 Linggong Road, Ganjingzi District, Dalian 116024, P.R. China

[b] Department of Materials Science and Engineering, Kyoto University, Yoshida-honmachi, Sakyo-ku, Kyoto 606-8501, Japan

[c] Elements Strategy Initiative for Structural Materials (ESISM), Kyoto University, Yoshida-honmachi, Sakyo-ku, Kyoto 606-8501, Japan

[d] Key Laboratory for Anisotropy and Texture of Materials (Ministry of Education), School of Materials Science and Engineering, Northeastern University, Shenyang 110819, China

[e] Department of Materials Science and Engineering, Yeungnam University, Gyeongbuk 38541,

Korea

[f] Department of Materials and Synchrotron Radiation Engineering, Graduate School of Engineering, University of Hyogo, Himeji 671-2280, Japan

[g] National Institute for Materials Science (NIMS), 1-2-1 Sengen, Tsukuba 305-0047, Japan

[h] Japan Synchrotron Radiation Research Institute (JASRI), Sayo-gun, Hyogo 679-5198, Japan

[i] Institute for Materials Chemistry and Engineering, Kyushu University, Kasuga, Fukuoka 816-8580, Japan

[j] Department of Materials Science and Engineering, Virginia Tech, Blacksburg, VA 24061, USA

***Corresponding authors**

Yu Bai (ybai@dlut.edu.cn); TEL: (+86)-411-84709458
Nobuhiro Tsuji (nobuhiro-tsuji@mtl.kyoto-u.ac.jp); TEL: (+81)-75-753-5462; FAX: (+81)-75-753-4978

Supplementary:

Influence of planer defects on dislocation density calculation

It has been recognized that the dislocation density in the order of 10^{15} m^{-2} could be obtained in heavily deformed polycrystalline metals and alloys. However, the dislocation densities in the present high-Mn steel shown in **Fig. 3** reached in the order of 10^{15} m^{-2} only after engineering tensile strains of 0.02 - 0.04. In principle, these dislocation densities were

evaluated from broadening of diffraction peaks, using the Equation (2). Local lattice distortion due to lattice defects introduced by plastic deformation changes the local diffraction condition. Accumulation of such changes of local diffraction conditions appears as peak broadening in the diffraction profile. Accordingly, not only dislocations but also planer defects, such as stacking faults and deformation twins, can cause broadening of X-ray diffraction peaks {Ungár, 2007}. However, it should be noted that stacking faults and deformation twins are reported to broaden the peak width at bottom parts of diffraction peaks {Balogh, 2006}, so that the influence of such planer defects on the value of FWHM using the peak width at the middle height of peaks is thought to be small. Furthermore, area fractions of deformation twins and number of deformation twins in unit area were relatively low around yielding, as discussed in **Fig. 9**. On this basis, we infer that the influence of planer defects, i.e. stacking faults and deformation twins, on peak broadening is relatively small at early stages of tensile deformation. Since the contribution of planer defects different from dislocations was not considered in the present analysis, the values of the present dislocation densities might be somehow overestimated. However, the difference in the change of dislocation density between the UFG specimen and the 4.5 μm grain-sized specimen, which corresponded to the change of the yielding behavior, could be clearly recognized from **Fig. 3**, suggested that the enhanced strain-hardening behavior was a characteristic feature in the UFG specimen, as well as the yielding stage characterized by the discontinuous yielding.

DIC analysis for local strain distributions around yielding

In order to understand the different yielding behavior occurring in the high-Mn steel specimens having different average grain sizes in a macroscopic scale, strain distributions in the tensile specimens during tensile tests were analyzed by the DIC technique. **Figure S1** shows stress-strain curves around yielding and corresponding local strain maps obtained by the DIC analysis in the specimen with a coarse grain size (d) of 15.4 μm and UFG specimen (d = 0.79 μm). The DIC maps shown in **Fig. S1** (b-f) and (h-l) represents distributions of local equivalent strains at different tensile strains (e = 0.007, 0.015, 0.023, 0.032 and 0.045) also indicated in the stress-strain curves (**Fig. S1** (a) and (g)) for the 15.4 μm grain-sized specimen and UFG specimen, respectively. The magnitude of local equivalent strain in the maps is expressed in different colors according to the key color bar shown in **Fig. S1**. The 15.4 μm grain-sized specimen showed typical continuous yielding (**Fig. S1** (a)), while the UFG specimen showed discontinuous yielding with yield-drop (**Fig. S1** (g)). Local strain distributions around yielding in the 15.4 μm grain-sized specimen were somehow heterogeneous within the gage part, but did not show particular patterns (**Fig. S1** (b-f)). On the other hand, local strain distributions in the UFG specimen showed characteristic features around macroscopic yielding. Before the upper yield point, the strain distribution within the gage part was homogeneous and the strains were quite low at everywhere (**Fig. S1** (h)). Just after the yield-drop, V-shape bands with higher local strains were clearly recognized at center

regions of the gage part in **Fig. S1** (i). The strains out of the bands were obviously lower than the strains within the bands, which was the difference from the 15.4 μm grain-sized specimen where the distribution of local strains was not perfectly homogeneous but plastic deformation happened everywhere in the gage part (**Fig. S1** (c, d)). With increasing the global tensile strain, the V-shaped regions with higher local strains in the UFG specimen expanded toward both sides in the gage part (**Fig. S1** (j-l)), maintaining nearly the same color (i.e., nearly the same strain value). The development of local strain distributions in the UFG specimen shown in **Fig. S1** (h-l) showed a characteristic of Lüders banding (Lüders deformation) typically observed in conventional carbon steels with BCC crystal structure {Gere, 1997}. It has been reported that the UFG Al {Kamikawa, 2009}, Cu {Tian, 2018} and IF steel {Gao, 2014} showed the Lüders deformation corresponding to nearly flat parts on their stress-strain curves after discontinuous yielding. The Lüders strain, i.e., the tensile strain at the end of the flat part on the stress-strain curve in typical Lüders deformation {Rooyen, 1971}, in the UFG Al, Cu and IF steel was very large and sometimes over 10%. Although such a flat part was not obviously observed on the stress-strain curves of the present high-Mn steel specimens having ultrafine grain sizes (**Fig. S1** (a)), it was confirmed that their deformation behavior around yielding was heterogeneous and localized in a manner of Lüders deformation. Because the maximum strain localization within the band, which usually corresponded to the Lüders strain, was only 0.042 in **Fig. S1** (k) and the strain localized band

propagated throughout the gage part quickly, the Lüders deformation was not clearly recognized on the stress-strain curves in the present UFG high-Mn steel. The less-clear flat part after discontinuous yielding in the UFG high-Mn steel is probably attributed to the large strain-hardening ability of this material.

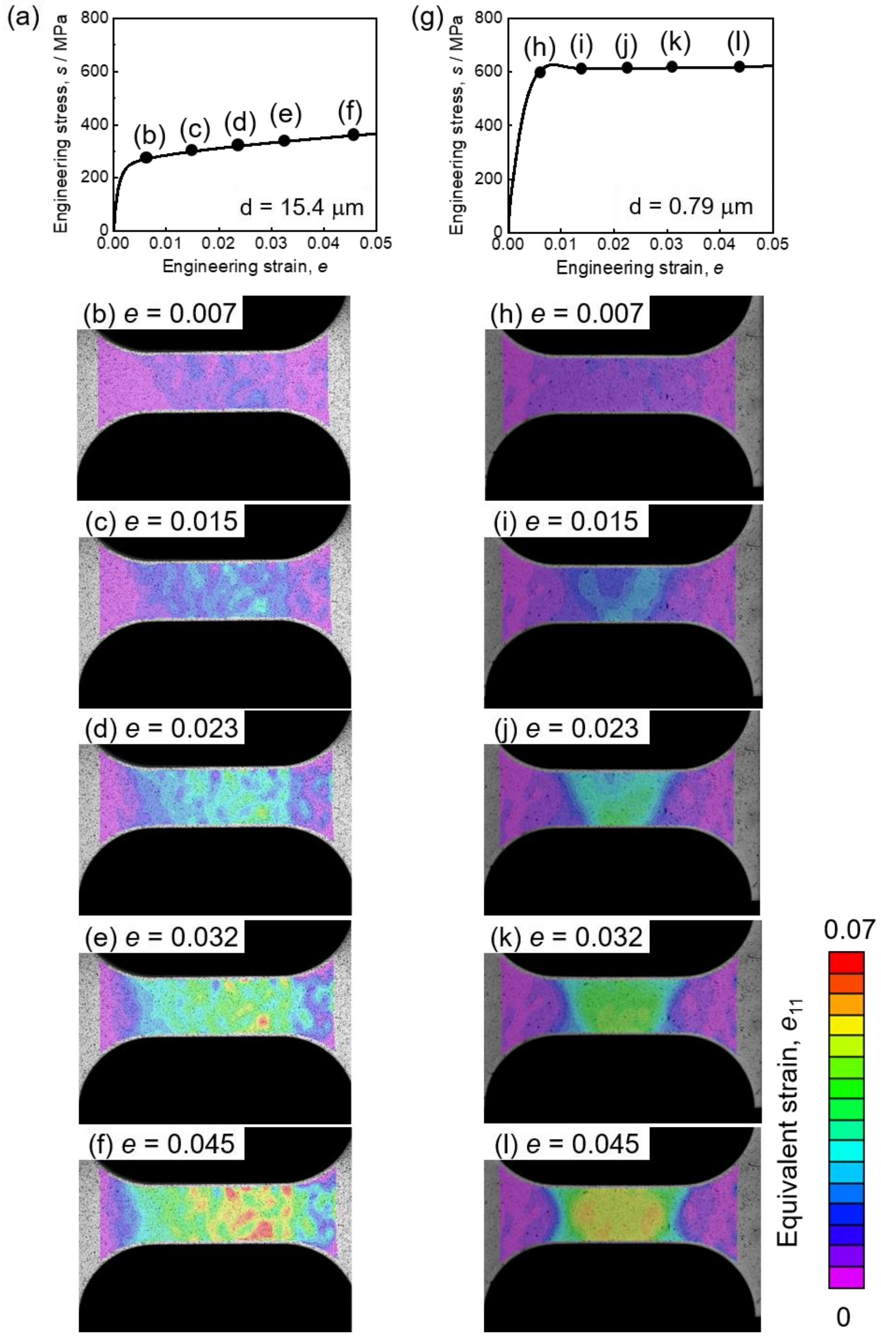

Figure S1 Engineering stress-strain curves around yielding of the 31Mn-3Al-3Si high-Mn steels with the grain sizes of (a) 15.4 μm and (g) 0.79 μm, and corresponding local equivalent strain maps in the tensile specimens obtained by the DIC analysis: (b-f) 15.4 μm and (h-l) 0.79 μm. (b-f) and (h-l) correspond to different strain stages indicated in (a) and (g), respectively.

References

1 Ungár, T. Characterization of nanocrystalline materials by X-ray line profile analysis. *J. Mater. Sci.* **42**, 1584-1593 (2007).Gere, J. M. & Timoshenko, S. P. *Mechanics of Materials* 835-842 (PWS Publishing, 1997).

2 Balogh, L., Ribárik, G. & Ungár, T. Stacking fault and twin boundaries in fcc crystals determined by x-ray diffraction profile analysis. *J. Appl. Phys.* **100**, 023512 (2006).

3 Gere, J. M. & Timoshenko, S. P. *Mechanics of Materials* 835-842 (PWS Publishing, 1997).

4 Kamikawa, N., Huang, X. X., Tsuji, N. & Hansen, N. Strengthening mechanisms in nanostructured high-purity aluminum deformed to high strain and annealed. *Acta Mater.* **57**, 4198-4208 (2009).

5 Tian, Y. Z. *et al.* Remarkable transitions of yield behavior and Lüders deformation in pure Cu by changing grain sizes. *Scr. Mater.* **142**, 88-91 (2018).

6 Gao, S. *et al.* Yielding behavior and its effect on uniform elongation of fine grained IF steel. *Mater. Trans.* **55**, 73-77 (2014).

7 Rooyen, G. T. Van Basic factors which influence the Lüders strain during discontinuous yielding. *Mater. Sci. Eng.* **7**, 37-48 (1971).
